# Everolimus-Based Therapy versus Chemotherapy among Patients with HR+/HER2− Metastatic Breast Cancer: Comparative Effectiveness from a Chart Review Study

**DOI:** 10.1155/2015/240750

**Published:** 2015-05-20

**Authors:** Nanxin Li, Yanni Hao, Jipan Xie, Peggy L. Lin, Valerie Koo, Erika Ohashi, Eric Q. Wu

**Affiliations:** ^1^Analysis Group, Inc., Boston, MA 02199, USA; ^2^Novartis Pharmaceuticals Corporation, East Hanover, NJ 07936, USA; ^3^Analysis Group, Inc., New York, NY 10020, USA

## Abstract

*Objective*. To compare the real-world effectiveness of everolimus-based therapy and chemotherapy in postmenopausal women with hormone-receptor-positive/human-epidermal-growth-factor-receptor-2-negative (HR+/HER2−) metastatic breast cancer (mBC).* Methods*. This retrospective chart review examined a nationwide sample of postmenopausal HR+/HER2− mBC women in community-based oncology practices. Patients received everolimus-based therapy or chemotherapy for mBC between 07/01/2012 and 04/15/2013, after failure of a non-steroidal aromatase inhibitor. Overall survival (OS), progression-free survival (PFS), and time on treatment (TOT) were compared using Kaplan-Meier analysis and Cox proportional hazards models adjusting for line of therapy and baseline characteristics.* Results*. 234 and 137 patients received everolimus-based therapy and chemotherapy. Patients treated with everolimus-based therapy tended to have less aggressive mBC than patients treated with chemotherapy. Multivariate-adjusted Cox models showed that everolimus-based therapy was associated with significantly longer OS [hazard ratio (HR) = 0.37, 95% confidence interval (CI): 0.22–0.63], PFS (HR = 0.70, 95% CI = 0.50–0.97), and TOT (HR = 0.34, 95% CI: 0.25–0.45) than chemotherapy. Adjusted comparative effectiveness results were generally consistent across lines of therapy.* Conclusion*. In this retrospective chart review of postmenopausal HR+/HER2− mBC patients, treatment with everolimus-based therapy was associated with longer OS, PFS, and TOT than chemotherapy.

## 1. **Introduction**

Breast cancer (BC) is the most common cancer in women worldwide [1]. Nearly 233,000 new cases were estimated to be diagnosed in 2014 in the United States, representing 14% of all new cancer cases [2]. Although metastatic BC (mBC) is diagnosed in only 5% of cases [2], nearly 30% of BC patients with earlier stage tumors eventually develop metastases [3]. This advanced disease is associated with worse prognosis than early stage BC, with 5-year survival rates around 25% [2]. Most BC samples overexpress hormone receptors (HR), including estrogen receptor (ER) and/or progesterone receptor (PR) [4, 5], whereas human epidermal growth factor receptor 2 (HER2) overexpression only occurs in 20–30% of cases [6]; thus, the most common BC subtype is HR+/HER2− [7]. Postmenopausal women, in particular, are more likely to have HR+/HER2− tumors, as HR overexpression increases with age [7].

The National Comprehensive Cancer Network (NCCN) [8] treatment guidelines for HR+/HER2− mBC recommend the use of endocrine therapy, particularly a nonsteroidal aromatase inhibitor (AI), as first-line treatment in postmenopausal women [8]. Since most patients eventually develop resistance to these therapies, the NCCN guidelines recommend another endocrine agent when the first therapy fails. After the failure of three sequential endocrine therapies, if symptomatic visceral disease is present or if the cancer is rapidly progressing or immediately life-threatening chemotherapy is recommended, either as monotherapy with an anthracycline, taxane, antimetabolite, or other microtubule inhibitors or as combination treatment [8]. However, observed real-world treatment patterns are not consistent with NCCN guidelines, showing that many HR+/HER2− mBC patients only receive one line of endocrine therapy before switching to chemotherapy for second-line treatment [9, 10]. Chemotherapy is often accompanied by serious treatment side effects (grade 3/4), some of which have severe impact on the patients' health-related quality-of-life (QOL) [11, 12]. Therefore, there is an unmet need for efficacious but more tolerable alternatives for the treatment of HR+/HER2− mBC.

A novel targeted agent, everolimus, was approved in July 2012 to be used in combination with endocrine therapy exemestane for the treatment of mBC in patients who failed nonsteroidal AI. The efficacy of everolimus/exemestane combinational therapy was demonstrated in the phase III, double-blind, randomized, BOLERO-2 trial with significantly improved progression-free survival (PFS) compared to exemestane monotherapy [13–15]. The efficacy of other everolimus-based therapies, such as combinational therapy of everolimus and tamoxifen, has also been examined in other clinical studies [16].

Currently, there is limited evidence regarding the comparative effectiveness of everolimus-based therapy and chemotherapy, two common treatment options for HR+/HER2− mBC after initial failure of nonsteroidal AI. Several recent studies presented in oncology conferences have indicated that everolimus/exemestane combinational therapy was associated with significantly longer survival compared to chemotherapies [17, 18], but these studies were based on small samples of patients [17] or physician surveys [18]. A network meta-analysis of previous mBC trials also found that everolimus/exemestane combinational therapy was associated with comparable or better PFS compared to some commonly-used chemotherapy; however, findings in clinical trial settings may not represent the real-world comparative effectiveness. In addition, this analysis also had inherent limitations due to indirect comparisons of treatments from different studies with heterogeneous designs and patient populations [19].

The present study aims to address this knowledge gap and to compare the real-world effectiveness of everolimus-based therapy versus chemotherapy in treating HR+/HER2− mBC patients in community-based oncology practices in the US. The everolimus-based therapy group includes everolimus monotherapy and combination therapy of everolimus and either endocrine therapy or chemotherapy; the chemotherapy group includes chemotherapy monotherapy, combinational therapy of chemotherapy agents, and combinational therapy of chemotherapy and endocrine therapy (excluding combination of chemotherapy and everolimus). The clinical outcomes of interest include time on treatment (TOT), overall survival (OS), and PFS.

## 2. **Data and Methods**

### 2.1. Data Source

Community-based oncologists/hematologists who treated postmenopausal women with HR+/HER2− stage IV mBC were invited from a nationwide online panel of over 9,500 oncologists/hematologists to participate in the chart review study. Physicians were eligible for participation if they had treated one or more postmenopausal HR+/HER2− mBC patients who met all the patient selection criteria described below. Each physician was asked to provide data for up to 10 patients, selected at random from their list of eligible patients. Stratified sampling was used to ensure sufficient sample size in each treatment group (everolimus-based therapy or chemotherapy) and by line of therapy (first line, second line, third line, and above). A standardized electronic case report form (eCRF) was developed to collect patient information through a secure online portal. The eCRF was extensively tested for logic and consistency and was pilot tested by three community based oncologists for clarity and understandability. All patient data were abstracted in an anonymous and nonidentifiable format. The study was approved by the New England Institutional Review Board (IRB). The identity of physicians was blind to the authors and study sponsor, and vice versa.

### 2.2. Patient Selection

Patient medical records were selected for abstraction if the patient was a postmenopausal woman who had BC recurrence or progression on or after a nonsteroidal AI in an adjuvant or metastatic setting and subsequently initiated an everolimus-based therapy or chemotherapy in any line of treatment for mBC between 07/01/2012 and 04/15/2013 (the first treatment initiated during this time period that met the aforementioned criteria was defined as the index therapy). Patients who received everolimus-based therapies before their index treatment were excluded from the current analysis for both treatment groups.

Furthermore, physicians were required to have access to their patients' mBC-related medical records from the first mBC diagnosis to the last follow-up (or death), while patients were required to not be enrolled in any clinical trials and to not have a history of primary malignancy of other nonbreast cancers (with the exception of nonmelanoma skin cancer and carcinoma in situ of the uterine cervix) within 3 years prior to the first mBC diagnosis date. The chart abstraction was completed in 09/2014.

### 2.3. Study Outcomes

Study outcomes included TOT, OS, and PFS. TOT was defined as the time from initiation of index therapy to either death or discontinuation of index therapy, whichever occurred first. Patients without recorded death or discontinuation of the index therapy were censored at the last follow-up date. OS was defined as the time from initiation of the index therapy to death from any cause. Patients without recorded death were censored at the last follow-up date. PFS was defined as time from initiation of index therapy to disease progression or death, whichever occurred first. Progression was determined by the participating physicians with radiographic evidence or tests, physical exams, or assessment of symptoms or through the use of other methods.

### 2.4. Statistical Analysis 

#### 2.4.1. Analysis of Baseline Characteristics

Patients' baseline characteristics at either the first mBC diagnosis or the initiation of index therapy were summarized. They included age, race, insurance type, disease status (*de novo*, recurrent with adjuvant endocrine therapy, recurrent without adjuvant endocrine therapy), adjusted Charlson Comorbidity Index (CCI) (excluding a score of 6 for metastatic cancer), Eastern Cooperative Oncology Group (ECOG) performance status, number and sites of metastases, physician-classified tumor volume, prior chemotherapy in the mBC setting, and time from initiation of last adjuvant endocrine therapy to the first mBC diagnosis. Patient baseline characteristics were compared between the everolimus-based therapy and chemotherapy groups using Wilcoxon rank-sum tests for continuous variables and chi-square tests for categorical variables.

#### 2.4.2. Analysis of Study Outcomes

Patients treated with everolimus-based therapy and chemotherapy were compared for all study outcomes (TOT, OS, and PFS) using Kaplan-Meier (K-M) analyses and Cox proportional hazards models. Unadjusted comparisons between everolimus-based therapy and chemotherapy included (1) K-M curves generated for each study outcome and log-rank tests; (2) median estimates obtained for patients who were not censored for TOT and PFS (e.g., for TOT, medians were assessed among those patients who had completed their index treatment); (3) Cox models used to compare each outcome using two approaches, one included treatment group assuming homogeneous comparative effectiveness across lines of therapy, and the other one included an interaction term between line of therapy and treatment group allowing heterogeneous comparative effectiveness across lines of therapy.

Adjusted comparisons between everolimus-based therapy and chemotherapy were conducted using multivariate Cox models, controlling for patient baseline characteristics including age, race, insurance type, index therapy line, disease status, adjusted CCI, sites of metastases, ECOG performance status, prior chemotherapy in the mBC setting, and time from initiation of last adjuvant endocrine therapy to first mBC diagnosis. Similar to the unadjusted Cox regression analyses, one set of models adjusted for treatment group and the other adjusted for an interaction term between the line of therapy and treatment group.

All analyses were performed using SAS version 9.3. Statistical significance was assessed at the 0.05 level. Chemotherapy was the reference group in all Cox regression models.

## 3. **Results**

### 3.1. Baseline Characteristics

A total of 234 patients were included for the everolimus-based therapy group and 137 for the chemotherapy group. Baseline characteristics comparisons were summarized in Table 1. Both groups had similar comorbidity burden, insurance coverage, ECOG performance status, prior chemotherapy in the mBC setting, and time from initiation of last adjuvant endocrine therapy to first mBC diagnosis. Patients treated with everolimus-based therapy were older (64 years versus 62 years, *P* = 0.050) and more likely to be Caucasian than patients treated with chemotherapy (64.1% versus 50.4%, *P* = 0.009). Compared to the chemotherapy group, everolimus-based therapy group had a lower proportion of liver, lung, and visceral metastases, a smaller number of metastatic sites, and a lower proportion of high-/medium-volume tumors (all *P* < 0.05). Overall, patients treated with everolimus-based therapy appeared to have less aggressive mBC than those treated with chemotherapy.

### 3.2. TOT

**Figure 1:**
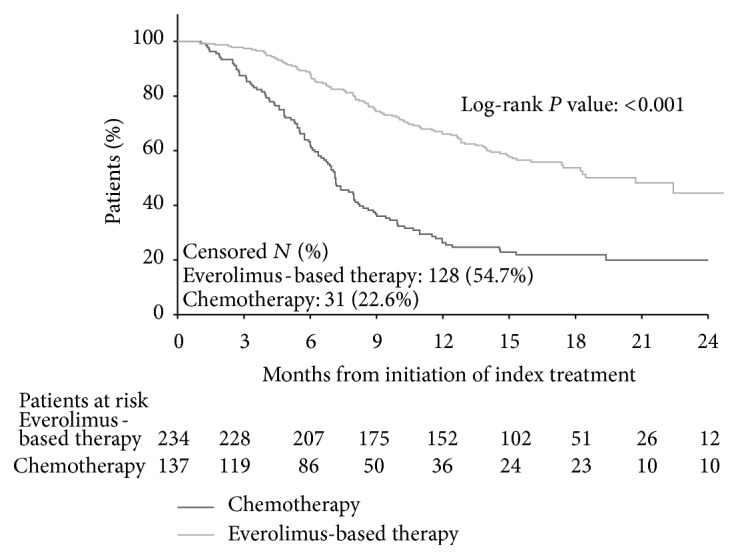
Comparison of time on treatment between everolimus-based therapy and chemotherapy.

K-M curves of TOT are shown in Figure 1. Everolimus-based therapy was associated with significantly longer TOT than chemotherapy (log-rank test *P* < 0.001; unadjusted hazard ratio (HR) = 0.36, 95% confidence interval (CI): 0.27–0.47, *P* < 0.001; Table 2). Median TOT among patients who completed their index treatment was 8.6 months for everolimus-based therapy patients and 6.1 months for chemotherapy patients. Multivariate-adjusted Cox regression results showed that TOT was significantly longer for everolimus-based therapy patients compared to chemotherapy patients (adjusted HR = 0.34, 95% CI: 0.25–0.45, *P* < 0.001; Table 2). When further adjusted by the interaction between line of therapy and treatment group, TOT was longer in patients who received everolimus-based therapy in all lines of therapy than patients who received chemotherapy in the same lines (adjusted first-line HR = 0.30, 95% CI: 0.20–0.46, *P* < 0.001; adjusted second-line HR = 0.30, 95% CI: 0.17–0.52, *P* < 0.001; adjusted third-line and above HR = 0.45, 95% CI: 0.26–0.78, *P* = 0.004; Table 3).

### 3.3. OS

**Figure 2:**
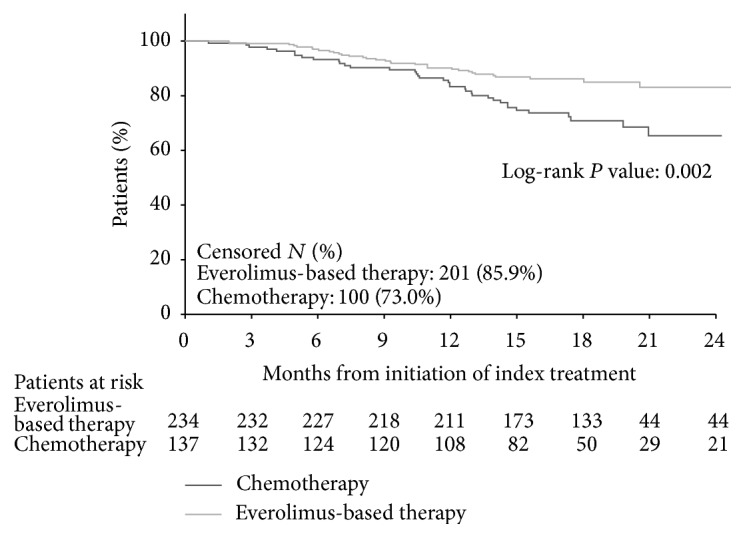
Comparison of overall survival between everolimus-based therapy and chemotherapy.

K-M curves of OS are shown in Figure 2. Everolimus-based therapy was associated with significantly longer OS than chemotherapy (log-rank test *P* = 0.002; unadjusted HR = 0.49, 95% CI: 0.30–0.78, *P* = 0.003; Table 2). Multivariate-adjusted Cox model results showed that OS was significantly longer for everolimus-based therapy patients compared to chemotherapy patients (adjusted HR = 0.37, 95% CI: 0.22–0.63, *P* < 0.001; Table 2). When further adjusted by the interaction between line of therapy and treatment group, OS was significantly longer in patients who received everolimus-based therapy in first-line or third-line and above than patients who received chemotherapy in the same lines (adjusted first-line HR = 0.35, 95% CI: 0.16–0.79, *P* = 0.011; adjusted third-line and above HR = 0.29, 95% CI: 0.12–0.75, *P* = 0.010; Table 3).

### 3.4. PFS

**Figure 3:**
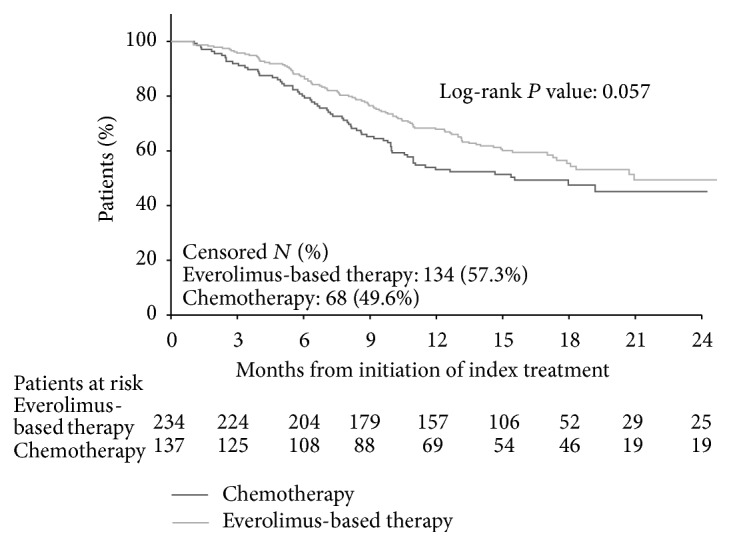
Comparison of progression-free survival between everolimus-based therapy and chemotherapy.

K-M curves of PFS are shown in Figure 3. Everolimus-based therapy was associated with numerically longer PFS than chemotherapy, although the difference was only marginally significant (log-rank test *P* = 0.057; unadjusted HR = 0.74, 95% CI: 0.55–1.01, *P* = 0.058; Table 2). Median PFS among patients who completed their index treatment was 8.5 months for everolimus-based therapy patients and 7.1 months for chemotherapy patients. Multivariate-adjusted Cox regression results showed that PFS was significantly longer for everolimus-based therapy patients compared to chemotherapy patients (adjusted HR = 0.70, 95% CI: 0.50–0.97, *P* = 0.033; Table 2). When further adjusted by the interaction between line of therapy and treatment group, PFS was longer in patients who received everolimus-based therapy in third-line and above than patients who received chemotherapy in third-line and above, although the difference was marginally significant (adjusted HR = 0.56, 95% CI: 0.30–1.02, *P* = 0.059; Table 3).

## 4. **Discussion**

For the treatment of HR+/HER2− mBC, the NCCN guidelines recommend three consecutive lines of endocrine therapy (including everolimus/exemestane combinational therapy for patients who meet the eligibility criteria for the BOLERO-2 trial) before chemotherapy [8]. However, real-world studies report that many patients start chemotherapy earlier [9, 10], possibly due to concerns about endocrine resistance or visceral symptoms [20]. As newer targeted therapies become available for HR+/HER2− mBC, evidence of the comparative effectiveness of these treatments versus chemotherapy is important for the decision-making process of physicians and payers. The current retrospective chart review showed that in HR+/HER2− postmenopausal women with mBC, patients receiving everolimus-based therapy tended to have less aggressive mBC, in particular visceral metastases, than patients receiving chemotherapy. Everolimus-based therapy was associated with significantly longer OS, PFS, and TOT than chemotherapy after adjusting for the observed baseline characteristics; and the findings were largely consistent across lines of therapy.

The present comparative effectiveness findings are consistent with recent studies showing that HR+/HER2− mBC patients treated with everolimus-based therapy tended to have better OS [17, 18] and PFS [19] than those treated with chemotherapy. For example, using a small sample of HR+/HER2− mBC patients, Pouget et al. showed that everolimus plus endocrine therapy resulted in significantly longer OS than chemotherapy for patients pretreated with two or fewer lines of therapies for mBC [17, 18]. Cope et al. (2013) conducted a network meta-analysis of available mBC clinical trials and concluded that despite differences in patient characteristics across studies, everolimus/exemestane combinational therapy was associated with the longer mean PFS until 20 months compared to commonly-used chemotherapies such as capecitabine, doxorubicin, paclitaxel, and vinorelbine [19]. Future head-to-head clinical trial evidence will help further assess the comparative efficacy of everolimus-based therapy compared to chemotherapy. A phase II BOLERO-6 trial [21] is actively recruiting patients and aims to compare the efficacy of chemotherapy (capecitabine monotherapy) with everolimus-based therapy in ER+ mBC patients after recurrence or progression on prior nonsteroidal AI. Primary findings of the study are expected in early 2016.

While chemotherapy is recommended for more aggressive cancers, for the majority of HR+/HER2− mBC patients—who present a more manageable course of disease—endocrine therapy presents a more favorable risk-benefit profile, particularly due to the treatment's similar efficacy but milder toxicity relative to chemotherapy [22]. The current NCCN guidelines recognize this and support subsequent treatment to prolong the benefits of endocrine therapies for as long as possible before initiating chemotherapy [8]. Together with recent studies [17, 18], the current findings suggest that everolimus-based therapy may be a more effective alternative to chemotherapy after initial failure of nonsteroidal AIs. Furthermore, previous studies have shown that while everolimus may result in some moderate toxicity [23, 24], the adverse events are generally manageable [25] and the patient's health-related QOL is similar to that of patients on endocrine therapy [26]. This may make everolimus more preferable over chemotherapy, which is often accompanied by severe tolerability issues that result in worse health-related QOL while on treatment [11, 12, 22]. The observed shorter TOT among patients treated with chemotherapy may be due to oncologists' preference of only prescribing a limited cycle of chemotherapy in order to avoid cumulative toxicity. Future studies can compare real-world safety outcomes between the two treatments to better inform treatment decisions.

The current study is subject to the limitations inherent to retrospective chart review studies. First, inherent to observation studies, the findings may be subject to bias if important confounding factors are not identified and adjusted for in the study's analyses [27, 28]. In the current multivariable analyses, we adjusted for patient characteristics commonly recorded in medical charts and known to be prognostic for outcomes in mBC. These included characteristics such as age, race, insurance type, index therapy line, disease status, CCI, sites of metastatic disease, ECOG, prior chemotherapy in the mBC setting, and time from initiation of the last adjuvant endocrine therapy to the first stage IV mBC diagnosis. However, if patients treated with everolimus-based therapy are healthier based on unobserved measures of disease severity or have better coping skills, the results are likely to be biased in favor of everolimus. Second, the frequency of patient follow-up could be different between the two treatment groups. The group with more frequent visits to oncologists was more likely to be identified to have an event (such as discontinuation and progression). Therefore, the results may be biased against such group. These limitations can only be addressed with a well-conducted randomized-controlled trial. Nonetheless, observational studies constitute a valuable and rich source of data, as they allow researchers to examine treatment effectiveness across patient groups in a large sample set and are directly reflective of (and applicable to) real-world clinical practice [29].

## 5. **Conclusion**

In this retrospective review of HR+/HER2− mBC patients from community-based oncology practices in the US, patients treated with everolimus-based therapy tended to have less aggressive mBC than patients treated with chemotherapy. After controlling for the observed baseline characteristics, everolimus-based therapy was associated with significantly longer OS, PFS, and TOT than chemotherapy. As this is an observational study, unobserved patient characteristics may affect study findings.

## Figures and Tables

**Table 1:** Comparison of patient baseline characteristics between everolimus-based therapy and chemotherapy.

Baseline characteristics^1^	Everolimus-based therapy	Chemotherapy	*P* value^†^
	*N* = 234	*N* = 137	
Age (years)			
Median (range)	64.0 (41.0, 89.0)	62.0 (38.0, 81.0)	0.050^∗^
Race/ethnicity, *n* (%)			
White	150 (64.1)	69 (50.4)	0.009^∗^
Non-white	84 (35.9)	68 (49.6)	
Insurance plan type, *n* (%)			
Commercial/private insurance	133 (56.8)	80 (58.4)	0.466
Medicare only	81 (34.6)	50 (36.5)	
Others	20 (8.5)	7 (5.1)	
Index therapy line			
First line	84 (35.9)	69 (50.4)	0.014^∗^
Second line	61 (26.1)	33 (24.1)	
Third line and above	89 (38.0)	35 (25.5)	
Adjusted CCI^1^			
Median (range)	0.0 (0.0, 5.0)	0.0 (0.0, 8.0)	0.172
Sites of metastatic disease, *n* (%)			
Bone	150 (64.1)	77 (56.2)	0.132
Liver	82 (35.0)	71 (51.8)	0.002^∗^
Lung	92 (39.3)	84 (61.3)	<0.001^*^
Visceral metastases	148 (63.2)	116 (84.7)	<0.001^∗^
Number of metastatic sites, *n* (%)			
1	111 (47.4)	24 (17.5)	<0.001^∗^
2	79 (33.8)	53 (38.7)	
3	36 (15.4)	47 (34.3)	
4	8 (3.4)	12 (8.8)	
5	0 (0.0)	1 (0.7)	
Physician assessed tumor volume			
High	18 (7.7)	29 (21.2)	<0.001^∗^
Medium	132 (56.4)	92 (67.2)	
Low	84 (35.9)	16 (11.7)	
ECOG performance status			
0—Asymptomatic	65 (27.8)	34 (24.8)	0.655
1—Symptomatic but completely ambulatory	100 (42.7)	68 (49.6)	
2—Symptomatic, <50% in bed during the day	27 (11.5)	12 (8.8)	
3—Symptomatic, >50% in bed, but not bedbound	4 (1.7)	1 (0.7)	
Not recorded in medical record	38 (16.2)	22 (16.1)	
Prior chemotherapy in mBC setting	52 (22.2)	23 (16.8)	0.209
Disease status			
Recurrent patients with adjuvant ET, *n* (%)	148 (63.2)	106 (77.4)	0.008^∗^
Recurrent patients without adjuvant ET, *n* (%)	37 (15.8)	9 (6.6)	
De novo, *n* (%)	49 (20.9)	22 (16.1)	
Months from initiation of last adjuvant endocrine therapy to the first stage IV mBC diagnosis			
Median (range)	17.9 (0.0, 149.6)	14.2 (0.0, 163.7)	0.458

^†^Statistical comparisons were conducted using Wilcoxon rank-sum tests for continuous variables and chi-square tests for categorical variables. ^∗^ *P* < 0.05.

Notes:

^1^The adjusted CCI calculated the comorbidity index excluding metastatic breast cancer (score of 6).

**Table 2:** Full models of univariate and multivariate-adjusted comparisons between everolimus-based therapy and chemotherapy.

Characteristic (reference group)	Time to discontinuation			Overall survival			Progression-free survival		
	HR	95% CI	*P* value	HR	95% CI	*P* value	HR	95% CI	*P* value
Everolimus-based therapy (chemotherapy) unadjusted	**0.36 **	**(0.27, 0.47)**	0.0000^∗^	**0.49 **	**(0.30, 0.78)**	0.0027^∗^	**0.74 **	**(0.55, 1.01)**	**0.0582**
Everolimus-based therapy (chemotherapy) adjusted	**0.34 **	**(0.25, 0.45)**	0.0000^∗^	**0.37 **	**(0.22, 0.63)**	0.0002^∗^	**0.70 **	**(0.50, 0.97)**	0.0326^∗^
Index therapy line (First line)								
Second line	1.11	(0.70, 1.75)	0.657	1.41	(0.65, 3.05)	0.380	1.26	(0.77, 2.07)	0.357
Third line and above	0.98	(0.61, 1.56)	0.918	1.07	(0.46, 2.50)	0.875	1.30	(0.78, 2.15)	0.314
Disease status (de novo)									
Recurrent with adjuvant ET	1.79	(1.11, 2.89)	0.018^∗^	1.46	(0.62, 3.44)	0.391	1.74	(1.04, 2.93)	0.036^∗^
Recurrent without adjuvant ET	0.63	(0.34, 1.17)	0.144	0.69	(0.24, 1.99)	0.489	0.47	(0.23, 0.98)	0.043^∗^
Age at index therapy initiation	1.00	(0.97, 1.03)	0.911	1.07	(1.02, 1.12)	0.006^∗^	1.01	(0.98, 1.04)	0.355
Race (all other races)									
White	0.68	(0.51, 0.91)	0.009^∗^	0.92	(0.55, 1.52)	0.739	0.77	(0.56, 1.06)	0.111
Insurance at mBC diagnosis (neither insurance)									
Private	1.79	(0.95, 3.39)	0.072	1.49	(0.44, 5.08)	0.525	2.36	(1.07, 5.21)	0.033^∗^
Medicare only	1.83	(0.91, 3.66)	0.089	1.04	(0.29, 3.81)	0.949	2.28	(0.99, 5.25)	0.053
CCI at index therapy initiation	1.00	(0.87, 1.14)	0.957	1.04	(0.85, 1.28)	0.713	0.95	(0.82, 1.12)	0.561
Sites of metastasis at index therapy initiation									
Bone	1.42	(1.06, 1.91)	0.020^∗^	1.28	(0.76, 2.13)	0.351	1.90	(1.34, 2.69)	0.000^∗^
Visceral	1.80	(1.10, 2.96)	0.020^∗^	1.36	(0.56, 3.27)	0.498	1.83	(1.08, 3.12)	0.025^∗^
Performance status at index therapy initiation (ECOG 0)									
ECOG 1	1.10	(0.76, 1.60)	0.600	1.78	(0.83, 3.81)	0.137	1.78	(1.14, 2.77)	0.011^∗^
ECOG 2	2.29	(1.36, 3.86)	0.002^∗^	4.73	(1.89, 11.84)	0.001^∗^	4.13	(2.31, 7.40)	0.000^∗^
ECOG 3	20.29	(7.05, 58.35)	0.000^∗^	264.30	(63.54, 1,099.46)	0.000^∗^	49.13	(16.74, 144.24)	0.000^∗^
None	1.28	(0.79, 2.05)	0.317	3.36	(1.33, 8.49)	0.011^∗^	2.10	(1.22, 3.62)	0.008^∗^
Previous chemotherapy treatment in mBC setting	1.39	(0.92, 2.11)	0.121	2.51	(1.22, 5.15)	0.012^∗^	1.21	(0.76, 1.92)	0.424
Duration from initiation of last adjuvant ET to mBC diagnosis	0.99	(0.99, 1.00)	0.008^∗^	1.00	(0.99, 1.01)	0.961	1.00	(0.99, 1.00)	0.173

^∗^ *P* < 0.05.

**Table 3:** Hazard ratios (HRs) comparing everolimus-based therapy and chemotherapy by line of therapy.

Everolimus-based therapy versus chemotherapy^1^	Time to discontinuation			Overall survival			Progression-free survival		
	HR	95% CI	*P* value	HR	95% CI	*P* value	HR	95% CI	*P* value
Unadjusted									
First line	0.32	(0.21, 0.49)	0.000^∗^	0.47	(0.22, 0.99)	0.048^∗^	0.87	(0.55, 1.39)	0.561
Second line	0.29	(0.17, 0.50)	0.000^∗^	0.53	(0.21, 1.29)	0.162	0.59	(0.32, 1.09)	0.093
Third line and above	0.51	(0.31, 0.85)	0.010^∗^	0.46	(0.20, 1.06)	0.070	0.71	(0.41, 1.24)	0.231
Multivariate-adjusted^2^									
First line	0.30	(0.20, 0.46)	0.000^∗^	0.35	(0.16, 0.79)	0.011^∗^	0.86	(0.53, 1.40)	0.553
Second line	0.30	(0.17, 0.52)	0.000^∗^	0.53	(0.20, 1.39)	0.195	0.61	(0.32, 1.17)	0.138
Third line and above	0.45	(0.26, 0.78)	0.004^∗^	0.29	(0.12, 0.75)	0.010^∗^	0.56	(0.30, 1.02)	0.059

^∗^ *P* < 0.05.

Notes:

^1^Chemotherapy was the reference group.

^2^The model adjusted for the following variables: age, line of therapy, adjusted CCI, sites of metastatic disease, ECOG performance status, and prior chemotherapy in the mBC setting at the index therapy initiation date. Insurance plan type, race, disease status, and months from initiation of last adjuvant endocrine therapy to the first stage IV mBC diagnosis were assessed at mBC diagnosis.
